# Pedagogy for Teaching Fire Safety through Design-Based Immersion of the National Building Code 2016 with Feedback from Students of Undergraduate Architecture

**DOI:** 10.1155/2023/4007123

**Published:** 2023-03-20

**Authors:** Raja Singh

**Affiliations:** ^1^Department of Architecture, School of Planning and Architecture, New Delhi, India; ^2^ISAC Centre for Built Environment Policy, Delhi NCR, India; ^3^Built Environment and Public Health Research Fellow, Tathatara Foundation, Bobbili, India

## Abstract

This study is based on the feedback from 3^rd^-year architectural undergraduate students at a leading architectural education college in India. An undergraduate degree in architecture in India leads to a professional license to practice as an architect in India. Fire safety is also a component of the architectural curriculum, but there are concerns worldwide that architecture colleges may somehow not give the impetus fire safety education may require. A studio-based, immersive pedagogy was created to make fire safety more relevant and easy to grasp for architecture students. This method used integrating the interventions from the country's fire code into the design using students' self-created design problems, with which they were familiar. This design-based immersive integration of the National Building Code 2016 and its fire provisions were tested in this study. The detailed course pedagogical structure has been presented. The study was tested using feedback from the students at the end of the semester using an 11-part questionnaire which 32 students answered in an anonymous mode. The results show an overall positive response where the students prefer a design-based integrated fire safety curriculum which introduces fire codes to the students in the applied format. This study paves the way for more replications of the studio design-based integration of fire codes into the curricula of architecture colleges. Further studies will require this technique to undergo further testing by involving practitioners who have undergone this pedagogy and testing the same in building projects.

## 1. Introduction

Fire safety for building projects was taught to the students in the third year of a five-year architecture undergraduate program. These students were at the Department of Architecture, School of Planning and Architecture, New Delhi. This was part of a subject dealing with building services, including air conditioning, mechanical mobility, and fire safety. This study takes feedback from students for a pedagogy used to teach students of 3^rd^-year Bachelor of Architecture [1]. This pedagogy integrates Part 4 of the National Building Code (NBC) 2016, dealing with fire and life safety [2] into the curriculum. The focus was on incorporating the code provisions through building design rather than only the basic theory, with students overlaying the fire provisions on the drawings of their existing academically introduced building design projects. The anonymised feedback was taken to check the effectiveness of the course from the students, and the importance of teaching fire safety through the design and integration of codes into buildings has been highlighted in this study.

### 1.1. Background

According to one study, fire protection may be neglected in architecture schools as it may be considered as an “engineering thing” [3]. In other studies, it is believed that the fire safety system may not always be considered seriously in the building design. The reasons attributed are lack of coordination between fire engineers and architects, difficulty and complexity of fire codes, and expensive fire installation costs [4]. Due to the lack of effective teaching in architecture schools, some studies further state that fire safety must be made a separate course and its teaching in separate modules may not be enough [5]. There are concerns in some countries that fire safety may not even be a part of the architectural curriculum in some institutions [6]. There has been a worldwide growth in fire safety engineering courses and a rise in remedial short courses based on technology. However, design teaching may remain a question. This may be due to the nascency in the industry of fire safety engineering for the perfect method of teaching fire safety engineering and checking the efficiency of the same [7]. In recent years, practitioners from Spain state that in architecture classrooms, the shift has been made from simply reading the legal requirements of fire to attempting to see the graphical or drawing-based representations of fire safety in building plans [8]. Fire safety is essential for architects as there are some building types or sizes for which only the architects must comply with the fire code, and there are no further checks. In case of further checks for buildings requiring those, there may be a hindrance that architects are believed to face when dealing with fire authorities, as they may assume their knowledge to be limited [9]. The basic requirement is the visual translation of the complexity of the fire codes that exist. This is possible by hands-on integration or immersion of the fire codes into design exercises and implementation of the codes in live building plans.

### 1.2. Need for the Study

There have been fires that cause damage to life and property in buildings across the world. Fire prevention is critical and must be integrated into the buildings. The course of architecture in the institution where the study was performed has minimal exposure to a module on fire that lasts one semester. This module is not entirely dedicated to fire but is given to building services, including the air-conditioning design and mechanical mobility, which leaves less time for fire safety integration into the design. There may be teaching on fire safety, but it is incidental in the architectural design courses, whereby the teachers of architectural design may teach as and when the students may need a solution to a particular problem dealing with fire safety integration with a design. In India, like the rest of the world, preconstruction building plan approvals also demand thorough integration of the NBC 2016 into the submission drawings. Architects in practice may depend upon fire engineers and consultants, but integrating codes into the design at the early stages of a building project may be necessary. The literature studies have shown neglect for fire safety teaching to architecture students, and this learning may need to be more design immersive. This article introduces a pedagogy that tries to bridge the possible gap in applied fire safety and its instruction to architecture students. This pedagogical technique also introduces the comprehensive integration of NBC 2016 into the architectural design school at the mid-undergraduate level, as we will see a gap in the knowledge of Part 4, Fire and Life Safety, from NBC 2016. The study is further strengthened by the feedback received from the students who underwent the course. The anonymity of the students ensured unbiased feedback. This study is also essential as there are not many, if not none, studies from the Indian perspective on this issue of design-based codal integration of fire safety into the curriculum of architecture educational institutes.

### 1.3. Aim of the Study

The aim of the study was to test a design-based approach of immersion of the fire component of NBC 2016 into the curriculum of 3^rd^-year architecture students through student feedback.

### 1.4. Objectives

1. To introduce a new pedagogy to teach fire safety to students of architecture at the undergraduate level
2. To base the pedagogy on a design immersion-based methodology focusing on design integration through applied fire safety principles
3. To see the familiarity of the students with the provisions of the NBC 2016, especially the provisions of Part 4: Fire and Life Safety
4. To integrate fire safety into the design by implementing the provisions of Part 4: Fire and Life Safety of the NBC 2016 into the curriculum

This article also shows an attempt made by the author to do the following:

1. Integrate Part 4 Life and Fire Safety of the NBC 2016 into the curriculum of third-year architecture students
2. Make the integration from merely reading the code to translating the same into an existing design project to get a completely immersive learning experience

### 1.5. About the National Building Code (NBC) 2016

NBC 2016 is also known as Special Publication No. 7, a compilation of the various standards developed by various committees of the Bureau of Indian Standards under the leadership of its Civil Engineering Department. It consists of 13 Parts, starting from Part 0 to Part 12. This is further divided into sections and subsections totalling up to 33 chapters [10]. This is a recommendation-based guideline that the Bureau of Indian Standards has provided, intending to provide a standardised building-level guideline for India [11]. Various state governments adapt in parts because land, buildings, and urban law are governed as a state legislature subject or a municipality-level subject in India. Of particular interest is Part 4 of the NBC 2016, which is titled Fire and Life Safety and deals with three major features, i.e., fire prevention, life safety, and fire protection. It has been adopted by many local building bye-laws to be followed as a mandatory provision with some customisation made from state to state, with Delhi, for example, making most parts mandatory to be followed, at least with a legal check on certain buildings of a particular size and use [12].

### 1.6. About the Course Taught

As per the syllabus, this course aims to acquaint students with systems for fire safety and codes relevant to them and incorporate the systems in the building design. The anticipated learning outcomes for the fire were that students could interpret and depict fire safety requirements in designs and drawings. This course is in line with the Council of Architecture (Minimum Standards of Architectural Education) Regulations, 2020 [13], made by the Council of Architecture under Section 45, read along with Section 21 of the Architects Act, 1972 [14]. This course was based on the premise that architecture may not be taught but is learnt and so is fire safety engineering, which should be taught through the “studio” environment. This is especially true for fire safety as there are unknown problems and unique solutions which may not be copy-paste for all designs as every design in the first place is unique [7].

In this course, immersive means a design studio-based approach [7] and does not mean the current connotation of immersive, which may mean virtual reality-based immersive learning technology [15]. The elective may not contain all the requirements of a full-time master's program in fire safety engineering [16], but it is a bridge between architecture studio and the applied portion for the building design. In the present scenario, the elective was part of a two-hour weekly lecture, but the instructor transformed the time into a studio-based exercise. The course was a two-credit program with an internal and external evaluation. In the external evaluation, the students were made to write an exam with questions that were set based on the application. A sample of the question put up in the exam is as follows: “What is FHC? Where are all these placed? Explain using a rough building plan (free hand, not to scale) and label the measurements (including distance between the two).” The FHC stands for fire hose cabinet. This enabled an application-based evaluation of the students' learning.

#### 1.6.1. The Pedagogical Technique of the Course

In the course, the step was sensitising students to significant fire disasters, which they could relate to from the newspaper articles. This was followed by an entire day trip to an underground transit station with state-of-the-art fire equipment. The steps after that were based on the classroom, which are the course methodology and are detailed in Table 1.

## 2. Methodology

The step-by-step methodology followed for this study is as follows:

1. An entire semester course was taught to students of 3^rd^ year Bachelor of Architecture at the School of Planning and Architecture, New Delhi. This course was a part of the combined subject to teach building services.
2. After completing the entire course, the feedback questionnaire was created to be circulated among the students.
3. The students were given the option to fill out the questionnaire anonymously, where they had the option of not filling in their name and class section. Eleven questions in total were asked.
4. The results of the questionnaire were collected, compiled, and reported.

The total number of students enrolled in the class was 86, of which 32 filled out the questionnaire. This means that the sample had a confidence level of 95% with a margin of error of 14%.

The questionnaire was in the form of an online Google Form titled “Anonymous Survey on Fire Class,” which had the following questions:

1. Name (optional) as students could fill the form anonymously.
2. Class and section (filing is optional)
3. Did you attend one or more fire safety in buildings classes this semester? (Yes/no/other)
4. Is asking students to make fire safety provisions on design sheets itself a good idea? (The 5-point Likert scale with one as “not great for learning” and five as “very good idea for learning”)
5. What has been your exposure to NBC 2016 before this class? (The 5-point Likert scale with 1 as “not familiar at all” and 5 as “very familiar”)
6. Should students be introduced to NBC at a stage earlier than third year? (Yes/no/other)
7. How easy was it for you to grasp the fire portion of the NBC 2016 by the end of the semester? (The 5-point Likert scale with 1 being “challenging” and 5 being “easy”)
8. Did the method of the class instruction make you understand the positive role of architects in fire safety in buildings? Rate. (The 5-point Likert scale with 1 being “not really” and 5 being “understand the positive role well”)
9. Was the teacher well prepared to sensitise about fire safety in buildings? (The 5-point Likert scale with 1 being “not prepared” and 5 being “well prepared”)
10. Should students be taught a lot of the fire theory or should be immersed into design integration? (The Likert scale with 1 being “theory based” and 5 being “design integration based”)
11. Any suggestions? Please write, if possible. It will help in improving the class curriculum in the future.

The answers to questions number 3 to 11 were compulsory to be answered. Question number 1 and 2 were purely optional to retain the survey's anonymity. Answer 11 was general feedback, mainly as a forum for students to present some difficulties they may have faced.

## 3. Results and Analysis

The results of the questionnaires are presented from question 4 onwards. In all of the Likert scale-based studies, an incremental Likert scale has been used, with 1 side usually being the negative value and 5 being the most positive. Points 2, 3, and 4 usually mean that 3 is neutral, 2 is more inclined towards value at 1, and 4 is more inclined towards value at 5.

**Figure 2:**
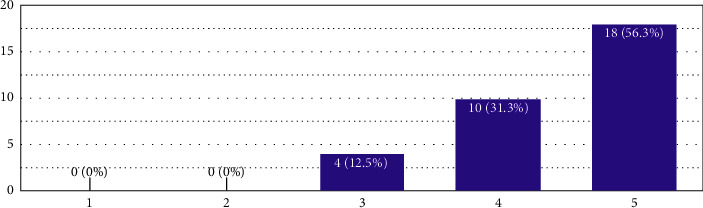
In the *X*-axis, 1 is to be read as “not great for learning,” 5 is to be read as “very good idea for learning,” and the remaining are the extrapolations in the middle as per the Likert scale. The *Y*-axis is the number of responses.

The interpretation of the average values can be made from Table 2:

The first question discussed was whether making fire safety provisions on design sheets was a good idea. The results are presented in Figure 2:

The second question asked was to judge the students' exposure to NBC 2016, which makes the reader understand the gap in familiarising important building codes for architecture students at the second half stage of their architecture professional curriculum. The average value, in this case, is 4.4, which means that the respondents agreed to the weight “5,” which means that asking students to make fire provisions in design sheets is a “very good idea for learning.”

**Figure 3:**
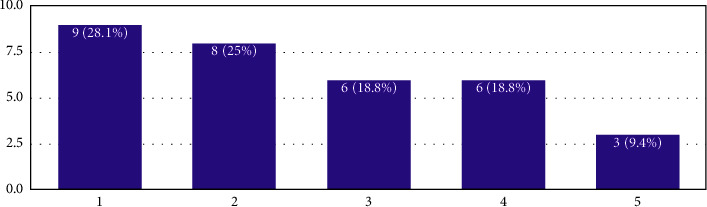
In this figure, on the *X*-axis, 1 represents “not familiar at all,” 5 represents “very familiar,” and the remaining are the extrapolations in the middle as per the Likert scale. The *Y*-axis is the number of responses.

In Figure 3, the average value was 2.56, which means that the respondents in the polarised scale answered as “2,” which is one weight lesser than “not familiar at all”.

**Figure 4:**
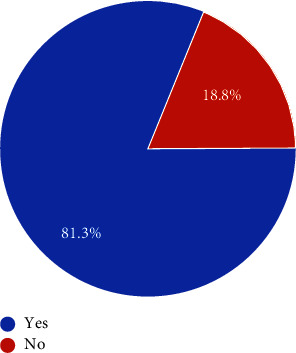
The answer to the question about the early introduction of the NBC 2016 to students of architecture. The blue is an affirmative “yes,” and the red is a “no.” The numbers represent percentages.

In Figure 4, the students were asked whether NBC 2016 should be introduced at a stage earlier than 3^rd^ year, to which the students answered in the positive, with 81.3% of students giving yes as an answer.

**Figure 5:**
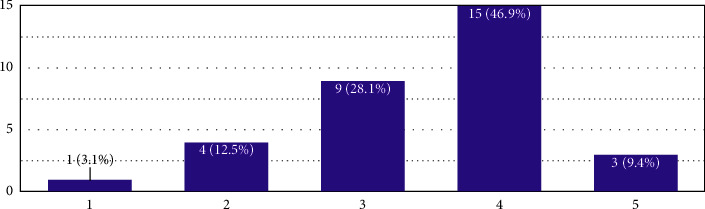
In the *X*-axis, “1” represents the polarised value “challenging,” “5” represents the polarised value “easy,” and the remaining are the extrapolations in the middle as per the Likert scale. The *Y*-axis represents the number of responses.

In Figure 5, the average value was 3.47, which categorises it in the weight “4,” which would mean that the respondents, on average, would agree instead of strongly agree for the easiness of grasping the fire portion of the NBC 2016 by the end of the teaching semester.

**Figure 6:**
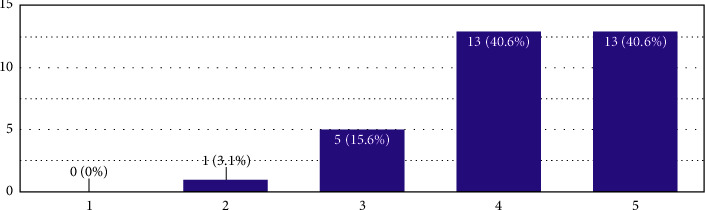
In the figure, “1” means “not really” whereas “5” represents “understand the positive role well;” the remaining are the extrapolations in the middle per the Likert scale. The *Y*-axis shows the number of responses.

In Figure 6, the average value came out to be 4.19, which categorises it be in “4,” which means that instead of strongly agreeing to “understand the role well” the average of the class responded as “agree,” which would mean that the respondents agree that the method of class instruction made them understand the positive role of architects in fire safety in buildings.

**Figure 7:**
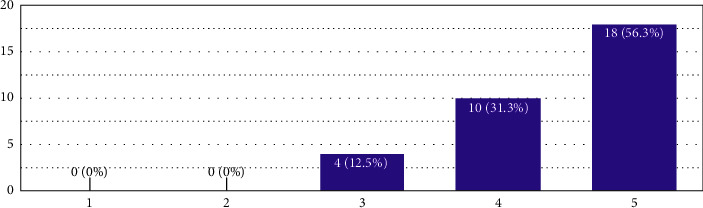
In the figure, “1” is “not prepared at all” and “5” is “well prepared”; the remaining are the extrapolations in the middle as per the Likert scale. The *Y*-axis shows the number of responses.

In Figure 7, the average value came out to be 4.44, which categorises it in the value of “5,” which means the respondents, on average, rated the teacher to be well prepared to sensitise students about fire safety in buildings.

This next question was fundamental to gauge the theory vs the design integration-based learning types that the students preferred for their comprehensive learning of fire safety in buildings.

**Figure 8:**
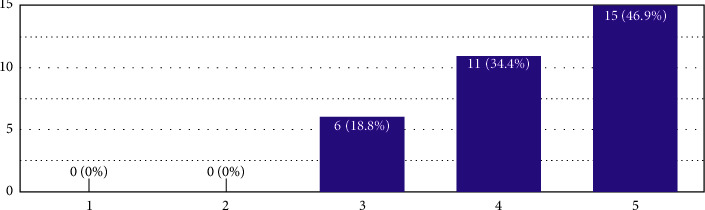
In this figure, the polarised entry 1 means “theory based” and 5 means “design intervention based;” the remaining are the extrapolations in the middle as per the Likert scale. The *Y*-axis shows the number of responses.

In Figure 8, the average value was 4.28, categorising it into the category of “5,” which means that the respondents wanted design intervention-based teaching instead of theory-based fire safety.

The results in totality can be summed up in the Table 3.

The study has only 32 students' feedback out of a class of 86. Though the sample size has been justified above, it needs to be pointed out that the students were given full anonymity and the choice of whether they wanted to complete the survey. The teacher did not compel any student to fill out the survey, and hence, the 32 responses are with the full willingness of the students and may be an accurate and unbiased response. It may be seen as a limitation of the study that the responses were low. Another limitation may be the assessment method used in the study, as only students' opinion was taken. Future research may involve taking feedback from alumni who become practitioners and provide inputs about putting the learning into real-world scenarios.

## 4. Discussion

As we have seen earlier, fire safety integration into the curriculum of architecture students needs extensive detail, vigour, and attention. Many fires happen in buildings where some architectural intervention or lack of it is the cause of the fire [18]. Some studies have even stated that architecture education does not focus enough on fire safety as needed [3, 6, 8, 18]. In India, there are many instances of fire where there are flouting of building codes or fire norms [17]. Many Indian building local bye-laws depend upon the provisions of the NBC 2016 for fire safety provisions. This makes the teaching of NBC 2016 very essential from the fire safety point of view and other building safety and design considerations. The results show that the awareness about NBC 2016 among students of architecture at the mid-degree level may not be adequate as there is evidence that the code should be introduced very early in the degree program.

Another crucial point is the visual nature of architectural education and the need to focus on simply theory-based learning to learning that integrates the applied portions of the building services segment to the drawings so that the process becomes seamless for students and they can relate to the subject strongly.

The case study of a building is an essential pedagogy technique as it is even a step higher than integration into the building design as the students would be able to see the physical manifestation of design intervention in reality. During the studio design, it was seen that many students made changes to their designs because of the newfound requirements they learnt from the fire codes. Some changes were fundamental, which required a significant change in the design.

Overall, it is crucial to consider that fire safety engineering as a curriculum is not new [7]. However, its focus has been the technology of fire safety. Furthermore, with buildings being a substantial place of fire occurrence, the design of buildings will require that fire be taught in the language of architects. The architectural methods of teaching technical modules using design-based studio learning have been hailed by scholars [7, 19]. This will require integrating fire safety interventions derived from fire codes into the architectural design studio at an early stage with a visual focus rather than only a theoretical and technical bent.

## 5. Conclusion

The study aimed to test a design-based approach of immersion of NBC 2016 into the curriculum of 3^rd^ year architecture students through student feedback. The results have shown a very positive response, with most students in favour of a design-based immersive approach where the principles of NBC 2016 are integrated into the design through overlays into the drawings of the building project familiar to the students. The study also shows the need for an early and more detailed interaction of students with the provisions of NBC 2016. Architectural educational institutes must increase the scope and intensity of fire safety education in schools of architecture. Another limitation may be the assessment method used in the study, as only students' opinion was taken. Future research may involve taking feedback from alumni who become practitioners and provide inputs about putting the learning into real-world scenarios. Future studies may also include a comparison of the curricula of architecture schools concerning the incorporation of the provisions of the NBC 2016 on fire safety.

The learning from this study may be helpful in the design-based inclusion of fire safety, especially for architectural colleges. With additional theoretical components, it may also serve well in other disciplines, especially the ones that deal with the technical part of fire extinguishing. This is because the inculcation of a design-based solution is essential as all buildings' designs are different, and the training in design-based solutions will equip a learner to look at fire safety not as a one-size-fits-all solution but as a customisable and iterative process.

## Figures and Tables

**Figure 1:**
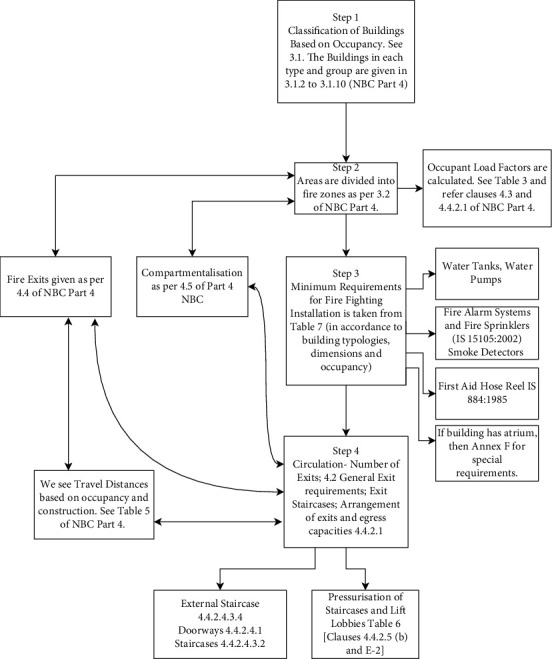
A corrected version of an easy graphical list prepared by a class student creates a list of actionable steps in order. These steps have been derived from NBC 2016, Part 4 Fire and Life Safety.

**Table 1:** The detailed step-by-step methodology for the semester-long course designed to teach fire safety to students of architecture.

S. no	Title of the course methodology step	Description of the course methodology step	Remarks
1	Case study of a model building with state-of-the-art fire safety provisions integrated into the design	A building with state-of-the-art fire safety was selected, and permission for a site visit was requested. The fire expert executed the site visit from the organisation which was visited. Students were made to touch and feel fire safety equipment and understand the various design interventions to make the building fire safe	The students were taken to an active mass transit transport station which was underground and heavily equipped with fire safety mechanisms

2	Sensitisation of the importance of fire safety in buildings by news-reported cases	The students were presented with multiple newspaper reports about fire incidences involving a loss of life and property. The students were made to study the highlighted points regarding the lapses in architectural design and noncompliance with fire codes which contributed to the gravity of loss in the fire incidences. Lapses may be a lack of ventilation or sealed windows[12]	The fire instances highlighted were those of the Mundka fire case in Delhi and other fire cases [17]

3	Reading through Part 4 of NBC 2016 and creating a step-by-step list of actionable steps	This is a significant step where a very bulky and text-based code was translated into graphical list-based actionable steps that the students could easily follow at the drawing board stage, and this will serve as a ready reckoner for the student	The students had to have a graphical list with arrows and the clause mentioned explicitly from the code. The graphical list sample prepared by a student is shown in Figure 1

4	Getting the drawings for a building project ready for future integration of fire provisions into it	This is a crucial step as in this, the students were asked to compulsorily get a studio project that they had designed in the past to have complete familiarity with the project's design. If not already carried out, integrating fire safety measures will be more effortless as the students can compensate for other design considerations and the building brief accordingly	The students chose the projects from their previous semester's design studio. The typology was public, and the students mostly had one of the following: (1)museums, (2)primary schools, (3)community centres, and (4)primary health care centres

5	Integrating the graphical list-based actionable steps into the existing building plans under exercise	The actionable steps collated in the list will be implemented in the design project selected. In order to fine-tune the broad principles, the students were to refer to the details in the code along with other related standards mentioned in the code to make an appropriate design. The students were to mark the fire safety interventions as an overlay on the building plans with proper notations and note the calculations in the drawing legend. Students were also provided with an existing fire plan of another building to see the notations and the symbols. The students were only to submit the overlay so that their inclination, as architecture students, to make drawings is further enforced	The students made a thorough effort to write down the calculations, step by step, as a legend in the drawing sheet, along with all the fire notations that were given by showing an actual sheet of another project made by a fire safety consultant

**Table 2:** The range of values to be used to interpret the weighted values with 0.8 as the difference between the two values.

Weight	Range of values	Interpretation generally in incremental order
1	1.00–1.80	Strongly disagree or otherwise mentioned in the caption of the figures below (SD)
2	1.81–2.60	Disagree or otherwise mentioned in the caption of the figures below (D)
3	2.61–3.40	Neutral or otherwise mentioned in the caption of the figures below (N)
4	3.41–4.20	Agree or otherwise mentioned in the caption of the figures below (A)
5	4.21–5.00	Strongly agree or otherwise mentioned in the caption of the figures below (SA)

**Table 3:** The summary of the results of the study.

S. no	Questions	Average weighted answer	Interpretation
1	Is asking students to make fire safety provisions on design sheets itself a good idea? (The 5-point Likert scale with 1 as “not great for learning” and 5 as “very good idea for learning”)	4.44	It means that asking students to make fire provisions in design sheets is a “very good idea for learning”

2	What has been your exposure to NBC 2016 before this class? (The 5-point Likert scale with 1 as “not familiar at all” and 5 as “very familiar”)	2.56	It means that the respondents in the polarised scale gave the answer as “2,” which is one weight lesser than “not familiar at all,” which would mean not familiar

3	Should students be introduced to NBC 2016 at a stage earlier than third year? (Yes/no/other)	Yes	81.3% of respondents stated a “yes”

4	How easy was it for you to grasp the fire portion of the NBC 2016 by the end of the semester? (The 5-point Likert scale with 1 being “challenging” and 5 being “easy”)	3.47	It means that the respondents, on average, would agree instead of strongly agree with the easiness of grasping the fire portion of the NBC 2016 by the end of the teaching semester

5	Did the method of the class instruction make you understand the positive role of architects in fire safety in buildings? Rate. (The 5-point Likert scale with 1 being “not really” and 5 being “understand the positive role well”)	4.19	It means that instead of strongly agreeing to “understand the role well,” the average of the class responded as “agree,” which would mean that the respondents agree that the method of class instruction made them understand the positive role of architects in fire safety in buildings

6	Was the teacher well prepared to sensitise about fire safety in buildings? (The 5-point Likert scale with 1 being “not prepared” and 5 being “well prepared”)	4.44	It means the respondents, on average, rated the teacher to be well prepared to sensitise students about fire safety in buildings

7	Should students be taught a lot of fire theory or should they be immersed in design integration? (The Likert scale with 1 being “theory based” and 5 being “design integration based”)	4.28	It means that the respondents, on average, wanted design intervention-based teaching instead of theory-based fire safety

## Data Availability

All the data used to support the findings of this study are included within the article.
